# Mathematics Anxiety: What Have We Learned in 60 Years?

**DOI:** 10.3389/fpsyg.2016.00508

**Published:** 2016-04-25

**Authors:** Ann Dowker, Amar Sarkar, Chung Yen Looi

**Affiliations:** Department of Experimental Psychology, University of OxfordOxford, UK

**Keywords:** mathematics anxiety, working memory, gender, stereotype threat, cognitive reappraisal, transcranial direct current stimulation (tDCS)

## Abstract

The construct of mathematics anxiety has been an important topic of study at least since the concept of “number anxiety” was introduced by Dreger and Aiken (1957), and has received increasing attention in recent years. This paper focuses on what research has revealed about mathematics anxiety in the last 60 years, and what still remains to be learned. We discuss what mathematics anxiety is; how distinct it is from other forms of anxiety; and how it relates to attitudes to mathematics. We discuss the relationships between mathematics anxiety and mathematics performance. We describe ways in which mathematics anxiety is measured, both by questionnaires, and by physiological measures. We discuss some possible factors in mathematics anxiety, including genetics, gender, age, and culture. Finally, we describe some research on treatment. We conclude with a brief discussion of what still needs to be learned.

Low achievement and low participation in mathematics are matters of concern in many countries; for example, recent concerns in the UK led to the establishment of the National Numeracy organization in 2012. This topic has received increasing focus in recent years, the ever-increasing importance of quantitative reasoning in a variety of educational and occupational situations, ranging from school examinations to management of personal finances.

Some aspects of mathematics appear to be cognitively difficult for many people to acquire; and some people have moderate or severe specific mathematical learning disabilities. But not all mathematical disabilities result from cognitive difficulties. A substantial number of children and adults have mathematics anxiety, which may severely disrupt their mathematical learning and performance, both by causing avoidance of mathematical activities and by overloading and disrupting working memory during mathematical tasks. On the whole, studies suggest that attitudes to mathematics tend to deteriorate with age during childhood and adolescence (Wigfield and Meece, 1988; Ma and Kishor, 1997), which has negative implications for mathematical development, mathematics education and adult engagement in mathematics-related activities. Also, while there are nowadays few gender differences in actual mathematical performance in countries that provide equal educational opportunity for boys and girls, females at all ages still tend to rate themselves lower in mathematics and to experience greater anxiety about mathematics than do males. It is important to understand children's and adults' attitudes and emotions with regard to mathematics if we are to remove important barriers to learning and progress in this subject.

Many studies over the years have indicated that many people have extremely negative attitudes to mathematics, sometimes amounting to severe anxiety (Hembree, 1990; Ashcraft, 2002; Maloney and Beilock, 2012). Mathematics anxiety has been defined as “a feeling of tension and anxiety that interferes with the manipulation of numbers and the solving of mathematical problems in … ordinary life and academic situations” (Richardson and Suinn, 1972).

Although, many studies treat mathematics anxiety as a single entity, it appears to consist of more than one component. Wigfield and Meece (1988) found two separate dimensions of mathematics anxiety in sixth graders and secondary school students and found two different dimensions: cognitive and affective, similar to those that had been previously identified in the area of test anxiety by Liebert and Morris (1967). The cognitive dimension, labeled as “worry,” refers to concern about one's performance and the consequences of failure, and the affective dimension, labeled as “emotionality” refers to nervousness and tension in testing situations and respective autonomic reactions (Liebert and Morris, 1967).

People have been expressing mathematics anxiety for centuries: the verse “Multiplication is vexation … and practice drives me mad” goes back at least to the sixteenth century. From a research perspective, the construct has been an important topic of study at least since the concept of “number anxiety” was introduced by Dreger and Aiken (1957), and has received increasing attention in recent years, in conjunction with the generally increased focus on mathematical performance.

Although, as will be discussed below, it is unclear to what extent mathematics anxiety causes mathematical difficulties, and to what extent mathematical difficulties and resulting experiences of failure cause mathematics anxiety; there is significant evidence that mathematics anxiety interferes with performance of mathematical tasks, especially those that require working memory. Moreover, whether a person likes or fears mathematics will clearly influence whether they take courses in mathematics beyond compulsory school-leaving age, and pursue careers that require mathematical knowledge (Chipman et al., 1992; Brown et al., 2008). Thus, mathematics anxiety is of great importance to the development and use of mathematical skills. It is also important in itself, as a cause of much stress and distress.

This paper will focus on what research has revealed about mathematics anxiety in the last 60 years, and what still remains to be learned. We will discuss what mathematics anxiety is, and how distinct it is from other forms of anxiety. We will discuss its relationship to attitudes to mathematics. We will then discuss the relationships between mathematics anxiety and mathematics performance and possible reasons for them. We will then discuss ways in which mathematics anxiety is measured, both by the commonest technique of questionnaires, and by physiological measures. We will then discuss some possible factors in mathematics anxiety, including genetics, gender, age, and culture. Finally and importantly, we will discuss some implications for treatment. We will conclude with a brief discussion of what still needs to be learned.

## Is mathematics anxiety separable from other forms of anxiety?

Though, as will be discussed below, mathematics anxiety is closely related to mathematical performance, it cannot be reduced just to a problem with mathematics. It seems to be as much an aspect of “anxiety” as an aspect of “mathematics.” Indeed, before assuming that mathematics anxiety is an entity in its own right, it is necessary to consider relationships between mathematics anxiety and other forms of anxiety, especially test anxiety, and general anxiety. Several studies suggest that mathematics anxiety is more closely related to other measures of anxiety, especially test anxiety, than to measures of academic ability and performance (Hembree, 1990; Ashcraft et al., 1998). Such studies typically show correlations of 0.3 and 0.5 between measures of mathematics anxiety and test anxiety.

Mathematics anxiety has also generally been found to correlate with measures of general anxiety; and it is indeed possible that this may serve as a background variable explaining some of the correlation between mathematics anxiety and test anxiety. For example, Hembree (1990) found a mean correlation of 0.35 between the MARS and a measure of general anxiety. In a behavioral genetic study, to be discussed in more detail below, Wang et al. (2014) obtained evidence that genetically based differences in general anxiety contribute to genetic differences in mathematics anxiety.

However, mathematics anxiety cannot be reduced to either test anxiety or general anxiety. Different measures of mathematics anxiety correlate more highly with one another (0.5–0.8) than with test anxiety or general anxiety (Dew et al., 1983; Hembree, 1990; review by Ashcraft and Ridley, 2005).

People may exhibit performance anxiety not only about tests and examinations, but about a variety of school subjects. Mathematics is usually assumed to elicit stronger emotional reactions, and especially anxiety, than most other academic subjects, but this assumption needs more research (Punaro and Reeve, 2012). Although, the general assumption is that people show much more anxiety and other negative attitudes toward mathematics than other academic subjects, there have not been many studies directly comparing attitudes to mathematics and other subjects.

Certainly anxiety toward other subjects exists, especially when performance in these subjects takes place in front of others. People with dyslexia have been found to exhibit anxiety about literacy (Carroll et al., 2005; Carroll and Iles, 2006). It is well-known, that foreign language learning and use, especially by adults, is often inhibited by anxiety (Horwitz et al., 1986; Cheng et al., 1999; Wu and Lin, 2014). Music students, and even successful musicians, often demonstrate music performance anxiety (Kenny, 2011).

Drawing also elicits performance anxiety and lack of confidence, and there is a decline in confidence with age, which in some ways parallels findings with regard to mathematics. Most young children enjoy drawing, and will often draw spontaneously. Many authors report that interest in drawing seems to decline in most children at or before the transition to secondary school, and many older children and adults will insist that they “can't draw,” even though they had drawn frequently and enthusiastically some years earlier (Cox, 1989; Thomas and Silk, 1990; Golomb, 2002; but see Burkitt et al., 2010 for somewhat conflicting findings).

Punaro and Reeve (2012) reported a study that directly compared mathematics and literacy anxiety in Australian 9-year-olds and related their anxiety to their actual academic abilities. Although, children expressed anxiety about difficult problems in both mathematics and literacy, worries were indeed greater for mathematics than literacy. Moreover, anxiety about mathematics was related to actual mathematics performance, whereas anxiety about literacy was not related to actual literacy performance. This study would suggest that although mathematics is not the only subject that elicits anxiety, anxiety may indeed be more severe, and possibly affect performance more, for mathematics than for other subjects.

## Mathematics anxiety and attitudes to mathematics

Attitudes to mathematics, even negative attitudes, cannot be equated with mathematics anxiety, as the former are based on motivational and cognitive factors, while anxiety is a specifically emotional factor. Nevertheless, attitude measures tend to correlate quite closely with mathematics anxiety. For example, Hembree (1990) found that in school pupils, mathematics anxiety showed a mean correlation of −0.73 with enjoyment of mathematics and −0.82 with confidence in mathematics. In college students, the equivalent mean correlations were a little lower than in schoolchildren, but still very high: −0.47 between mathematics anxiety and enjoyment of mathematics, and −0.65 between mathematics anxiety and confidence in mathematics.

Mathematics anxiety seems to be particularly related to self-rating with regard to mathematics. People who think that they are bad at mathematics are more likely to be anxious. Most studies indicate a negative relationship between mathematics self-concept and mathematics anxiety (Hembree, 1990; Pajares and Miller, 1994; Jain and Dowson, 2009; Goetz et al., 2010; Hoffman, 2010).

However, as most of these studies are correlational rather than longitudinal, it is hard once again to establish the direction of causation: does anxiety lead to a lack of confidence in one's own mathematical ability, or does a lack of confidence in one's mathematical ability make one more anxious? Ahmed et al. (2012) carried out a longitudinal study of 495 seventh-grade pupils, who completed self-report measures of both anxiety and self-concept three times over a school year. Structural equation modeling suggested that each characteristic influenced the other over time, but that the effect of self-concept on subsequent anxiety was significantly greater than the effect of anxiety on subsequent self-concept. The details of the results should be taken with some caution, because although the study was longitudinal, it was over a relatively short period (one school year) and also a different pattern might be seen among younger or older children. However, it provides evidence that the relationship between mathematics anxiety and mathematics self-concept is reciprocal: each influences the other.

A closely related construct is self-efficacy. Ashcraft and Rudig (2012) adapted Bandura's (1977) definition of self-efficacy to the topic of mathematics, stating that “self-efficacy is an individual's confidence in his or her ability to perform mathematics and is thought to directly impact the choice to engage in, expend effort on, and persist in pursuing mathematics” (p. 249). It is not precisely the same construct as self-rating, as it includes beliefs about the ability to improve in mathematics, and to take control of one's learning, rather than just about one's current performance; but there is of course significant overlap between the constructs. Studies have demonstrated an inverse relationship between self-efficacy and math anxiety (Cooper and Robinson, 1991; Lee, 2009).

Attitudes to mathematics also involve conceptualization of what mathematics is, and it is possible that this is relevant to mathematics anxiety. Many people seem to regard mathematics only as school-taught arithmetic, and may not consider other cultural practices involving numbers as mathematics (Harris, 1997). Also, people may not recognize that arithmetical ability (even without considering other aspects of mathematics) is made up of many components, not just a single unitary ability (Dowker, 2005). This can risk their assumption that if they have difficulty with one component, they must be globally “bad at maths,” thus increasing the risk of mathematics anxiety.

Most studies of mathematics anxiety have not differentiated between different components of mathematics, and it is likely that some components would elicit more anxiety than others and that the correlations between anxiety about different components might not always be very high. Indeed, studies which have looked separately at statistics anxiety and (general) mathematics anxiety in undergraduates have suggested that the two should be seen as separate constructs, and differ in important ways. For example, as will be discussed in the Section Gender and Mathematics Anxiety, most studies suggest that females show more mathematics anxiety than males, but there are no gender differences in statistics anxiety (Baloğlu, 2004).

## Prevalence of mathematics anxiety

Estimates of the prevalence of mathematics anxiety vary quite widely, and are of course likely to be dependent on the populations being sampled, on the measures used (though many of the studies involve similar measures), and, perhaps especially, on what criteria are used to categorize people as “mathematics anxious.” Most measures of mathematics anxiety assess scores on continuous measures, and there is no clear criterion for how severe the anxiety must be for individuals to be labeled as high in mathematics anxiety.

Richardson and Suinn (1972) estimate that 11% of university students show high enough levels of mathematics anxiety to be in need of counseling. Betz (1978) concluded that about 68% of students enrolled in mathematics classes experience high mathematics anxiety. Ashcraft and Moore (2009) estimated that 17% of the population have high levels of mathematics anxiety. Johnston-Wilder et al. (2014) found that about 30% of a group of apprentices showed high mathematics anxiety, with a further 18% affected to a lesser degree. Chinn (2009) suggested the far lower figure of 2–6% of secondary school pupils in England, which may simply indicate the use of an unusually strict criterion for defining pupils as having high mathematics anxiety. There is no doubt, even when taking the lowest estimates, that it is a very significant problem.

## Relationships between mathematics anxiety and mathematics performance

Numerous studies have shown that emotional factors may play a large part in mathematical performance, with mathematics anxiety playing a particularly large role (McLeod, 1992; Ma and Kishor, 1997; Ho et al., 2000; Miller and Bichsel, 2004; Baloğlu and Koçak, 2006). Mathematics anxiety scores correlate negatively with scores on tests of mathematical aptitude and achievement, while usually showing no significant correlation with verbal aptitude and achievement.

One possible reason for the negative association between mathematics anxiety and actual performance is that people who have higher levels of math anxiety are more likely to avoid activities and situations that involve mathematics. Thus, they have less practice (Ashcraft, 2002), which is in itself likely to reduce their fluency and their future mathematical learning.

Mathematics anxiety might also influence performance more directly, by overloading working memory (Ashcraft et al., 1998). Anxious people are likely to have intrusive thoughts about how badly they are doing, which may distract attention from the task or problem at hand and overload working memory resources. It has been found in many studies over the years that general anxiety as a trait is associated with working memory deficits (Mandler and Sarason, 1952; Eysenck and Calvo, 1992; Fox, 1992; Berggren and Derakhshan, 2013). It would appear likely that if anxiety affects working memory, it would have a particularly strong effect on arithmetic, as working memory has been found in many studies to be strongly associated with arithmetical performance, especially in tasks that involve multi-digit arithmetic and/or involve carrying (e.g., Hitch, 1978; Fuerst and Hitch, 2000; Gathercole and Pickering, 2000; Swanson and Sachse-Lee, 2001; Caviola et al., 2012). Thus, the load that mathematics anxiety and associated ruminations place on working memory could be a plausible explanation for decrements in mathematical performance.

Ashcraft and Kirk (2001) found that people with high maths anxiety demonstrated smaller working memory spans than people with less maths anxiety, especially in tasks that required calculation. In particular, they were much slower and made many more errors than others in tasks where they had to do mental addition at the same time as keeping numbers in memory.

DeCaro et al. (2010) asked adult participants to work out verbally based and spatially based mathematics problems in either low-pressure or high-pressure testing situations. Performance on problems that relied heavily on verbal WM resources was less accurate under high-pressure than under low-pressure tests. Performance on spatially based problems that do not rely heavily on verbal WM was not affected by pressure. Asking some individuals to focus on the problem steps by talking aloud helped to reduce pressure-induced worries and eliminated pressure's negative impact on performance.

While Ashcraft's theory emphasizes the ways in which mathematics anxiety impairs mathematical performance, some researchers such as Núñez-Peña and Suárez-Pellicioni (2014) put more emphasis on how pre-existing mathematical difficulties might cause or increase mathematics anxiety. Poor mathematical attainment may lead to mathematics anxiety, as a result of repeated experiences of failure.

Indeed, it appears that mathematics anxiety is associated not only with performance in high-level calculation skills that require the use of working memory resources, but also with much more basic numerical skills. For example, Maloney et al. (2011) gave high mathematics-anxious (HMA) and low mathematics-anxious (LMA) individuals two variants of the symbolic numerical comparison task. In two experiments, a numerical distance by mathematics anxiety (MA) interaction was obtained, demonstrating that the effect of numerical distance on response times was larger for HMA than for LMA individuals. The authors suggest that HMA individuals have less precise representations of numerical magnitude than their LMA peers; and that this may be primary, and precede the mathematics anxiety. In other words, mathematics anxiety may be associated with low-level numerical deficits that compromise the development of higher-level mathematical skills. Núñez-Peña and Suárez-Pellicioni (2014) also found that people with HMA showed a larger distance effect as well as a larger size effect (longer reaction times to comparisons involving larger numbers) than LMA individuals. Maloney and Beilock (2012) proposed that mathematics anxiety is likely to be due *both* to pre-existing difficulties in mathematical cognition *and* to social factors, e.g., exposure to teachers who themselves suffer from mathematics anxiety. Additionally, they proposed that those with initial mathematical difficulties are also likely to be more vulnerable to the negative social influences; and that this may create a vicious circle.

Studies of the relationship between mathematics anxiety and performance also need to take into account that, as stated at the beginning of this paper, mathematics anxiety consists of different components, often termed “cognitive” and “affective.” The cognitive and affective dimensions seem to be differently related to achievement in mathematics. For example, in sixth graders and secondary school students, the affective dimension of math anxiety has found to be more strongly negatively correlated with achievement than the cognitive dimension (Wigfield and Meece, 1988; Ho et al., 2000). It also needs to be remembered that, even before considering the non-numerical aspects of mathematics, arithmetic itself is not a single entity, but is made up of many components (Dowker, 2005).

## Assessments of mathematics anxiety

So far, we have been discussing mathematics anxiety without much reference to the methods used for studying it. However, in order to study mathematics anxiety, it is necessary to find suitable ways of assessing and measuring it. Most measures for assessing mathematics anxiety involve questionnaires and rating scales, and are predominantly used with adolescents and adults. The first such questionnaire to our knowledge is that of Dreger and Aiken (1957); and subsequent well-known examples include the Mathematics Anxiety Research Scale or MARS (Richardson and Suinn, 1972) and the Fennema–Sherman Mathematics Attitude Scales (Fennema and Sherman, 1976).

Some questionnaires, mainly including pictorial rating scales, have since been developed for use with primary school children; e.g., the Mathematics Attitude and Anxiety Questionnaire (Thomas and Dowker, 2000; Krinzinger et al., 2007; Dowker et al., 2012) and the Children's Attitude to Math Scale (James, 2013).

The reliability of mathematics anxiety questionnaires has generally been found to be good, whether measured through inter-rater reliability, test-retest reliability or internal consistency. The test whose psychometric properties have been most frequently assessed is the MARS, in its original form and in various adaptations, and it has been consistently found to be highly reliable (e.g., Plake and Parker, 1982; Suinn et al., 1972; Levitt and Hutton, 1984; Suinn and Winston, 2003; Hopko, 2003).

Good reliability has also been found for other mathematics anxiety measures such as Betz's (1978) Mathematics Anxiety Scale (Dew et al., 1984; Pajares and Urban, 1996) and the Fennema–Sherman scales (Mulhern and Rae, 1998). The mathematics anxiety scales developed specifically for children have also been found to have good reliability, including Thomas and Dowker's (2000) Mathematics Anxiety Questionnaire (Krinzinger et al., 2007); James' (2013) Children's Anxiety in Math Scale; and the scale developed by Vukovic et al. (2013).

Thus, it is unlikely that any ambiguous or conflicting results in different studies are likely to be due to unreliability of the measures. However, there are potential problems with questionnaire measures as such. In particular, a potential problem with questionnaire measures is that, like all self-report measures, they may be influenced both by the accuracy of respondents' self-perceptions and by their truthfulness in reporting. There are some studies that have attempted to combat this problem by using physiological measures of anxiety when exposed to mathematical stimuli: e.g., heart rate and skin conductance (Dew et al., 1984); cortisol secretion (Pletzer et al., 2010; Mattarella-Micke et al., 2011) and especially brain imaging measures ranging from EEG recordings (Núñez-Peña and Suárez-Pellicioni, 2014, 2015); to functional MRI (Lyons and Beilock, 2012b; Young et al., 2012; Pletzer et al., 2015).

## Physiological measures: cortisol secretion

Cortisol secretion is a response to stress (Hellhammer et al., 2009), and therefore might be expected to be higher in people with high levels of mathematics anxiety when presented with mathematical stimuli or activities. Studies do indeed support this view, as well as giving some clues about the interactions between mathematics anxiety and other characteristics.

Pletzer et al. (2010) investigated people's changes in cortisol level in response to the stress of a statistics examination, and the relationship between these changes and their actual examination performance. They were also assessed on a questionnaire measure of mathematics anxiety (a version of the MARS) and on tests of magnitude judgements and arithmetic. With a few exceptions who showed other patterns, most participants either showed an increase in cortisol from the basal level just before the examination, and a decrease afterwards, or a decrease in cortisol from the basal level both before and after the examination. Neither absolute levels or cortisol nor patterns of change in cortisol production correlated with the MARS, with the arithmetical tests, or with performance in the examination itself. However, the cortisol response to the examination did influence the association of other predictor variables and statistics performance. Mathematics anxiety and arithmetic abilities predicted statistics performance significantly in the group who showed an increase in cortisol production before the examination with a subsequent decrease, but not in the group that showed a consistent decrease.

Mattarella-Micke et al. (2011) measured cortisol secretion levels just before and after participants were presented with challenging mathematics problems. They also assessed their working memory. The performance of individuals with low working memory scores was not associated with mathematics anxiety or cortisol secretion. For people with higher working memory scores, those with high mathematics anxiety showed a negative relationship between cortisol secretion and mathematics performance, while those with low mathematics anxiety showed a positive relationship between cortisol secretion and mathematics performance.

Thus, in the studies carried out so far, the relationship between mathematics anxiety and cortisol response are not absolutely straightforward. It appears that the cortisol secretion profile modulates the relationship between mathematics anxiety and mathematics performance, while mathematics anxiety modulates the relationship between cortisol and performance. Thus, there are modulatory relationships between these measures, which are well worth studying further; but no evidence as yet that cortisol response is a good indicator of mathematics anxiety, or should replace traditional questionnaires.

## Physiological measures: what can measures of brain function tell us about mathematics anxiety?

Attempts at physiological measures of mathematics anxiety have more commonly involved some form of recording of brain function. Dehaene (1997, p. 235) argues that the neuroscience of mathematics can and must involve emotional factors: “…(C)erebral function is not confined to the cold transformation of information according to logical rules. If we are to understand how mathematics can become the subject of so much passion or hatred, we have to grant as much attention to the computations of emotion as to the syntax of reason.” It is, however, only quite recently that we have had the ability to carry out functional brain imaging with sufficient numbers of participants to be able to examine correlations between individual differences in brain function and individual differences in behavioral characteristics. It is even more recently that we have been able to apply functional brain imaging to children.

It is important to remember that finding neural correlates of behavioral characteristics does not mean that the brain characteristics are *causing* the behavioral characteristics. They are at least as likely to be *reflecting* the behavioral characteristics. Nevertheless, examining brain-based correlates of mathematics anxiety may give us some clues as to the cognitive characteristics involved, even if it does not tell anything about the direction of causation. They may also give us ways of assessing mathematics anxiety without needing to rely on self-report measures.

## Physiological measures: EEG/ERP

Núñez-Peña and Suárez-Pellicioni (2014, 2015) carried out both ERP and behavioral measures of numerical processing in people with high and low mathematics anxiety as measured on the MARS questionnaire. In a magnitude comparison test, people with high mathematics anxiety had slower reaction times and showed larger size and distance effects than those with low mathematics anxiety. ERP measures showed that those with high mathematics anxiety showed higher amplitude in frontal areas for both the size and distance effects than did those with low mathematics anxiety: a component which has been proposed to be associated with numerical processing. They also looked at two-digit addition in people with high and low mathematics anxiety. They were presented with correct and incorrect answers to such problems, and asked to say whether each answer was right or wrong. Participants with high mathematics anxiety were significantly slower and less accurate than those with low mathematics anxiety. ERP analysis showed that people with high mathematics anxiety showed a P2 component of larger amplitude than did people with low mathematics anxiety. This component had been previously found to be associated with devoting attentional resources to emotionally negative stimuli. Thus, the studies suggest that people with high mathematics anxiety may be devoting extra attentional resources to their worries, possibly at the expense of task performance, though the direction of causation cannot be determined from a correlational study.

## Physiological measures: functional MRI

There has been much evidence that stress affects the activation levels of regions of the prefrontal cortex, possibly interfering with the working memory functions associated with this area (Qin et al., 2009). These effects have been shown to be greater in people with high levels of general anxiety as a trait. For example, Bishop (2009) found that, even in the absence of threat stimuli, people with high trait anxiety showed less prefrontal activation in attentional control tasks than people with lower trait anxiety, and this was associated with less efficient performance. Basten et al. (2012) found that high trait anxiety was associated with high activation of the right dorsolateral prefrontal cortex (dLPFC) and left inferior frontal sulcus, which are generally found to be implicated in the goal-directed control of attention, and with strong deactivation of the rostral-ventral anterior cingulate cortex, a key region in the brain's default-mode network. The authors suggested that these activation patterns were likely to be associated with inefficient manipulations in working memory.

Lyons and Beilock (2012a) carried out functional brain imaging studies with adults with high and low mathematics anxiety. The individuals with high mathematics anxiety tended to show less activity in the frontal and parietal areas in anticipating and carrying out mathematical tasks than did less anxious individuals. They also did less well in the mathematical tasks. However, there was a subgroup, that did show strong activation of these areas when anticipating a mathematics task, and these individuals performed much better than those who did not show such activation, and almost as well as those with low mathematics anxiety. This group of individuals also showed high activation during the mathematics task, not so much of the parietal and other cortical areas associated with arithmetic, but of subcortical areas associated with motivation and assessment of risk and reward. The authors suggested that the deficit in performance of individuals with high mathematics anxiety might be determined by their response and interpretation of their anxiety response, instead of the magnitude of those anxiety response or their mathematics skills *per se*.

Pletzer et al. (2015) carried out an fMRI study of two groups of people, matched for their mathematical performance on tests of magnitude judgment and arithmetic, but differing in levels of mathematics anxiety, as measured by a version of the MARS. Eighteen participants scored high and 18 low on the measure of mathematics anxiety. They underwent fMRI when carrying out two numerical tasks: number comparison and number bisection. For comparison, they were also given brief non-numerical cognitive tasks involving verbal reasoning and mental rotation. The groups did not differ in their brain activation patterns for the non-numerical tasks. In the numerical tasks, they did not differ with regard to the activation of areas known to be involved in number processing, such as the intraparietal sulcus (similar to findings of Lyons and Beilock, 2012a,b) suggesting that performance deficits of high mathematics anxious individuals were unlikely to be due to lower mathematics skills; but the group with high mathematics anxiety showed more activity in other areas of the brain, especially frontal areas associated with inhibition. This suggests that processing efficiency may be impaired in people with high mathematics anxiety, requiring more effort to inhibit incorrect responses. The differences seemed to occur specifically for items that required magnitude processing, and were not found for items that involved multiplication and could readily be solved by fact retrieval.

Recently, functional brain imaging studies have indicated that 7- to 9-year-old children are already showing some of the same neural correlates of mathematics anxiety as adults. Young et al. (2012) carried out a functional MRI study with 7- to 9-year-old children, and found that mathematics anxiety was associated with high levels of activity in right amygdala regions that are involved in processing negative emotions and reduced activity in posterior parietal and dorsolateral prefrontal cortex regions associated with mathematical problem-solving (the latter finding was in contrast to Pletzer et al., 2015, Lyons and Beilock, 2012a,b who found no activation differences in these areas). Children with high mathematics anxiety also showed greater functional connectivity between the amygdala and areas in the ventromedial prefrontal cortex that are associated with negative activity was also positively correlated with task activity in two subcortical regions: the right caudate nucleus and left hippocampus, both of which are known to be involved in memory processes. Crucially, these brain activity differences were mainly found, not during the actual mathematics task, but during the cue that preceded it (similar to Lyons and Beilock, 2012b). Thus, the control processes that influence whether mathematics anxiety will inhibit performance seem to occur at the time of anticipation of the mathematics task, rather than during the task itself.

These studies have led to some interesting proposals about the most effective timing of cognitive treatments for mathematics anxiety. In particular, Lyons and Beilock (2012b, p. 2108) have proposed, on the basis of the above-mentioned brain-imaging studies and their own findings (greater activation in areas associated with visceral threat detection and pain perception with higher mathematics anxiety before but not during mathematics performance), that “emotional control processes that act early on the arousal of negative affective responses (e.g., reappraisal) are more effective at mitigating these responses and limiting concomitant performance decrements than explicit suppression of these responses later in the affective process.” As we shall see, this has implications for treatments.

## Factors that influence mathematics anxiety: genetics

So far, we have been discussing the nature and assessment of mathematics anxiety, without much reference to the factors that influence it. One potential factor that has been investigated is genetics. Wang et al. (2014) carried out behavioral genetic studies of mathematics anxiety in a sample of 514 twelve-year-old twin pairs. They were given the Elementary Students version of the MARS as a measure of mathematics anxiety; the Spence Children's Anxiety Scale as a measure of test anxiety; a mathematical problem solving subtest of the Woodcock-Johnson III Tests of Achievement; and a reading comprehension test from the Woodcock Reading Mastery Test. Mathematics anxiety correlated significantly with general anxiety, and also correlated negatively with both mathematical problem solving and reading comprehension, while general anxiety did not correlate significantly with either academic measure. Univariate and multivariate behavioral genetic modeling indicated that genetic factors accounted for about 40% of the variance in mathematics anxiety, with most of the rest being explained by non-shared environmental factors.

It is unlikely that there are genetic factors specific to mathematics anxiety. Rather, the multivariate analyses suggested that mathematics anxiety was influenced by the genetic and environmental risk factors involved in general anxiety, and the genetic factors involved in mathematical problem solving. Thus, mathematics anxiety may result from a combination of negative experiences with mathematics, and predisposing genetic risk factors associated with both mathematical cognition and general anxiety.

## Gender and mathematics anxiety

One of the factors that has received most study with regard to mathematics anxiety is that of gender. Much recent research indicates that males and females, in countries that provide equal education for both genders, show little or no difference in actual mathematical performance (Spelke, 2005). However, they do indicate that females tend to rate themselves lower and to express more anxiety about mathematics (Wigfield and Meece, 1988; Hembree, 1990; Else-Quest et al., 2010; Devine et al., 2012), though such differences are not huge (Hyde, 2005). Most studies suggest such gender differences only develop at adolescence, and that primary school children do not exhibit gender differences in mathematics anxiety (Dowker et al., 2012; Wu et al., 2012; Harari et al., 2013) though even in the younger age group boys often rate themselves higher in mathematics than girls do (Dowker et al., 2012). This increased anxiety may come from several sources, including exposure to gender stereotypes, and the influence and social transmission of anxiety by female teachers who are themselves anxious about mathematics (Beilock et al., 2010).

It may also be related to more general differences in anxiety between males and females. Many studies indicate that females tend to show higher levels of trait anxiety and the closely related trait of Neuroticism than males (e.g., Feingold, 1994; Costa et al., 2001; Chapman et al., 2007) and show higher prevalence of clinical anxiety disorders (McLean et al., 2011). They have been found to show greater anxiety than males even in subjects where their actual performance tends to be higher than that of males, such as foreign language learning (Park and French, 2013).

Also, males tend to show more confidence and rate themselves higher in a number of domains than females do (e.g., Beyer, 1990; Beyer and Bowden, 1997; Jakobsson et al., 2013). Thus, it is not surprising that this should also apply to mathematics, and, given the associations between anxiety and self-rating, that it might contribute to gender differences in mathematics anxiety.

However, there is some evidence that gender differences in mathematics anxiety cannot be reduced to gender differences in general academic self-confidence or in test anxiety. Devine et al. (2012) found that mathematics anxiety has an effect on mathematics performance, even after controlling for general test anxiety, in girls but not in boys. They asked 433 British secondary school children in school years 7, 8, and 10 (11-to 15-year-olds) to complete mental mathematics tests and Mathematics Anxiety and Test Anxiety questionnaires. Boys and girls did not differ in mathematics performance; but girls had both higher mathematics anxiety and higher test anxiety. Both girls and boys showed a positive correlation between mathematics anxiety and test anxiety and a negative correlation between mathematics anxiety and mathematics performance. Both boys and girls showed a negative correlation between mathematics anxiety and mathematics performance. However, regression analyses showed that for boys, this relationship disappeared after controlling for general test anxiety. Only girls continued to show an independent relationship between mathematics anxiety and mathematics performance.

By contrast, Hembree (1990) suggested that math anxiety is more negatively related to achievement in males than in females, and some other studies suggested that there are no gender differences in the relationship between mathematics anxiety and performance (Meece et al., 1990; Ma, 1999; Wu et al., 2012). However, most such studies have not controlled for general test anxiety. Gender effects on the relationship between mathematics anxiety and performance may also depend on whether one is examining the cognitive or affective component of mathematics anxiety, and on what aspects of mathematics are involved. Indeed, Miller and Bichsel (2004) found that mathematics anxiety was more related to basic mathematics scores in males, but to applied mathematics scores in females. More research is needed as to what influences gender differences in both mathematics anxiety itself, and in its influence on performance.

It is unlikely that such gender differences are the result of gender differences in working memory, as on the whole, studies show relatively few gender differences in working memory (Robert and Savoie, 2006) though some studies suggest that males may be better at visuo-spatial working memory and females at verbal working memory (Robert and Savoie, 2006). Intriguingly, Ganley and Vasilyeva (2014) carried out a mediation analysis that suggested that mathematics anxiety seemed to affect visuo-spatial working memory more in female than male college students, and that this led to a greater decrement in mathematics performance. However, since other studies suggest that mathematics anxiety affects verbal more than visuo-spatial working memory (DeCaro et al., 2010), there is still much room for further research here.

One possible explanation for greater mathematics anxiety in females than males is *stereotype threat*. Stereotype threat occurs in situations where people feel at risk of confirming a negative stereotype about a group to which they belong. In the domain of mathematics anxiety, this usually refers to females being reminded of the stereotype that males are better at mathematics than females, though it can also occur with regard to other stereotypes. For example, Aronson et al. (1999) found that white American men performed less well in mathematics when they were told that Asians tend to perform better in mathematics than white people, than when they were not exposed to this stereotype.

Most of the studies of the effects of stereotype threat on mathematics anxiety are somewhat indirect: they indicate that mathematics performance is worse when people are exposed to stereotype threat, but do not usually include direct measures of mathematics anxiety. While one likely explanation for the effects of stereotype threat is that it increases mathematics anxiety, there are other possibilities: e.g., that participants choose to conform to social expectations. This caution must be borne in mind when considering the evidence about the effects of stereotype threat on performance.

Schmader (2002) and Beilock et al. (2007) found that women performed less well on an arithmetic task if they were told that the researchers were studying why women do more poorly than men. Beilock et al. (2007) noted that, as is often found in studies of mathematics anxiety, the effect only occurred for problems that required the significant use of working memory resources.

Johns et al. (2005) gave participants a mathematics test under three conditions: one without any reference to gender stereotypes; one where they were told that the researchers were studying reasons why women performed less well in mathematics; and one where they were exposed to the same gender stereotype, but also taught explicitly about the nature of stereotype threat in this context, and how it could increase women's anxiety when doing mathematics. Females performed less well than men in the condition where the gender stereotype was presented without explanation, but there were no gender differences either in the condition where no gender stereotype was presented *or* in the condition where they were taught explicitly about the stereotype threat.

However, the effect of stereotype threat is not always found, especially in children. Ganley et al. (2013) carried out three studies with a total sample of 931 school children ranging from fourth to twelfth grade, and using several different methods from the implicit to the highly explicit to induce stereotype threat. There was no evidence of any effect of stereotype threat on girls' performance in any of these studies. It may be that stereotype threat only exerts an influence in very specific circumstances, or on the other hand that it always occurs and exerts an influence under all circumstances, so that the experimental manipulations exerted no additional effect. It may also be that the importance of stereotype threat has been overestimated at least with regard to children; or that the effects were greater in the past than now, due to changes in social attitudes.

Moreover, it may be that gender stereotypes are affecting not so much mathematics anxiety itself as self-perceptions of mathematics anxiety. Goetz and colleagues gave secondary school pupils questionnaires about mathematics anxiety as a *trait*, and also about their anxiety as a *state* during a mathematics class (Goetz et al., 2013; Bieg et al., 2015). Both boys and girls tended to report higher trait anxiety than state anxiety, but girls did so to a much greater extent. Girls reported higher trait anxiety than boys in both studies, but higher state anxiety only in one of the studies. One possible conclusion that girls do not in fact experience so much more mathematics anxiety than boys, but that due to gender stereotypes they *expect* to experience more mathematics anxiety, and this in itself may discourage them from pursuing mathematics activities and courses.

## Factors that affect mathematics anxiety: age

On the whole, mathematics anxiety appears to increase with age during childhood. Most studies suggest that severe mathematics anxiety is uncommon in young children, though some researchers have found significant mathematics anxiety even among early primary school children (Wu et al., 2012). This apparent increase in mathematics anxiety with age is consistent with findings that show that other attitudes to mathematics change with age. Unfortunately, they tend to deteriorate as children get older (Ma and Kishor, 1997; Dowker, 2005; Mata et al., 2012). Blatchford (1996) found that two-thirds of 11-years-olds rate mathematics as their favorite subject, but that few 16-year-olds do so. Some studies suggest that the deterioration of attitudes begins even before the end of primary school (Wigfield and Meece, 1988).

There are a number of reasons why mathematics anxiety might increase with age: some relating more to the “anxiety” and some more to the “mathematics.” One reason is that general anxiety appears to increase with age during childhood and adolescence could also reflect increases in tendency to general anxiety. For example, it is generally found that the onset of clinical anxiety disorders peaks in early adolescence (Kiessler et al., 2005) though it is possible that such disorders in younger children are under-diagnosed due to lack of clear and appropriate diagnostic methods (Egger and Angold, 2006). It may be that a factor such as increasing intolerance of uncertainty or increasing awareness of social comparison is leading to both increased general anxiety and to increased mathematics anxiety in particular.

Reasons more specifically relating to mathematics may include exposure to other people's negative attitudes to mathematics; to social stereotypes, for example about the general difficulty of mathematics or about supposed gender differences in mathematics; to experiences of failure or the threat of it; and/or to changes in the content of mathematics itself. Arithmetic with larger numbers that make greater demands on working memory, and more abstract non-numerical aspects of mathematics, may arouse more anxiety than the possibly more accessible aspects of mathematics encountered by younger children.

Moreover, the relationships between attitudes and performance may change with age. A meta-analysis by Ma and Kishor (1997) indicated that the relationship between attitudes and performance increases with age. Some studies suggest that among young children, performance is not significantly related to anxiety (Cain-Caston, 1993; Krinzinger et al., 2009; Dowker et al., 2012; Haase et al., 2012), but is more related to liking for mathematics and especially to self-rating. However, different studies give conflicting results; and some studies do show a significant relationship between anxiety and performance in young children (Dossey et al., 1988; Newstead, 1998; Wu et al., 2012; Ramirez et al., 2013; Vukovic et al., 2013).

There are at least three possible explanations for the conflicting findings. One is that the results may vary according to the aspect of mathematics anxiety that is being studied. Studies that base their measures on Richardson and Suinn (1972). Mathematics Rating Scale (MARS) or MARS-Elementary (Suinn et al., 1988) have tended to show such a relationship even in young children (Wu et al., 2012; Vukovic et al., 2013), and this could reflect the fact that such measures tend to focus on the affective dimension of mathematics anxiety. Those that have used the Mathematics Anxiety Questionnaire (MAQ) developed by Thomas and Dowker (2000) have tended not to show such a relationship in younger children (Krinzinger et al., 2007, 2009; Dowker et al., 2012; Haase et al., 2012; Wood et al., 2012), which could reflect the fact that this measure places more emphasis on the cognitive (“worry”) aspect of mathematics. The few studies that have included both dimensions of mathematics anxiety have suggested that performance in young children is related to the affective but not to the cognitive dimension (Harari et al., 2013), whereas studies of older children and adults suggest that performance is related to both, but is more strongly related to the affective dimension (Wigfield and Meece, 1988; Ho et al., 2000). More research is needed on how the relationship changes with age between performance and different components of mathematics anxiety.

A second explanation is that mathematics anxiety becomes more closely related to mathematics performance because of changes in working memory. Working memory of course increases with age in childhood (Henry, 2012), which could affect the relationship between anxiety and performance. One study does suggest that the relationship between anxiety and performance is greater in children with higher than lower levels of working memory. Vukovic et al. (2013) carried out a longitudinal study of 113 children, who were followed up from second to third grade. Mathematics anxiety was measured by items from the MARS-Elementary and from Wigfield and Meece's (1988) MAQ. Mathematics anxiety was negatively related to performance in calculation but not geometry. It was also negatively correlated with pupils' improvement from second to third grade, but only for children with higher levels of working memory. This is at first sight surprising given that working memory is generally positively correlated with mathematical performance, and especially in view of the theory that mathematics anxiety impedes performance by overloading working memory. We would suggest that a likely explanation is that among younger elementary school children, only those with high levels of working memory are already using mathematical strategies that depend significantly on working memory, and that therefore these may be the children whose progress is most impeded by mathematics anxiety. This could be one explanation for mathematics anxiety being more correlated with performance more in older than in younger children.

A third possible explanation is cultural. The studies that do show a relationship between mathematics anxiety and achievement among young children tend to be from the USA, though this could of course be a coincidence, and there are at present no obvious reasons why the relationship should be stronger in the USA than elsewhere. Nevertheless, there is evidence more generally for cultural influences on mathematics anxiety.

## Culture, nationality, and mathematics anxiety

Some aspects of attitudes to mathematics seem to be common to many countries and cultures: e.g., the tendency for young children to like mathematics, and for attitudes to deteriorate with age (Ma and Kishor, 1997; Dowker, 2005). However, different countries differ not only in actual mathematics performance, but also in liking mathematics; in whether mathematics is attributed more to ability or effort; and how much importance is attributed to mathematics (Stevenson et al., 1990; Askew et al., 2010).

Some of these differences could affect mathematics anxiety, though the direction is not completely predictable. Children in high-achieving countries could be low in mathematics anxiety because they are doing well (and/or may do well because they are not impeded by mathematics anxiety). On the other hand, they could be high in mathematics anxiety, because such countries often attach high importance to mathematics and to academic achievement in general, making failure more threatening; and because such children are likely to be comparing themselves with high-achieving peers, rather than with lower-achieving children in other countries. Lee (2009) investigated mathematics anxiety scores in a variety of countries and found that the relationship between a country's overall mathematics achievement level, and the average level of mathematics anxiety among children in that country, was not consistent. Children in high-achieving Asian countries, such as Korea and Japan, tended to demonstrate high mathematics anxiety; while those in high-achieving Western European countries, such as Finland, the Netherlands, Liechtenstein, and Switzerland tended to demonstrate low mathematics anxiety. At present, the reason for these differences is not clear. They may be related to the fact that pressure to do well in examinations is probably significantly greater in Asian countries (e.g., Tan and Yates, 2011). They could also be related to some as yet undetermined specific aspects of the educational systems or curricula.

Another possible reason could involve cultural or ethnic differences either in willingness to admit to mathematics anxiety, or in the nature of the relationship between mathematics anxiety and mathematics performance. Several studies have suggested that ethnic minority students express more positive attitudes to mathematics than white pupils both in the USA (Catsambis, 1994; Lubienski, 2002) and in the UK (National Audit Office, 2008), which did not conform to actual differences in performance. However, the meta-analysis of Ma (1999) showed no ethnic differences with regard to the relationship between anxiety and performance.

There is overwhelming evidence that both the socio-economic status of individuals and the economic position of countries have a very large influence on mathematical participation and achievement (e.g., Chiu and Xihua, 2008), However, there has been little research specifically on the influence of socio-economic status on mathematics anxiety or attitudes to mathematics; and the research that has been done does not suggest a very strong SES effect on these variables (Jadjewski, 2011).

## Potential treatments of mathematics anxiety

Research has already told us a lot about the nature of emotions and attitudes toward mathematics. So far, it tells us less about how such attitudes can be modified, and how mathematics anxiety may be treated, or, ideally, prevented. It is likely that early interventions for children with mathematical difficulties may go some way toward preventing a vicious spiral, where mathematical difficulties cause anxiety, which causes further difficulties with mathematics. Parents and teachers could attempt to model positive attitudes to mathematics and avoid expressing negative ones to children. This may, however, be difficult if the parents or teachers are themselves highly anxious about mathematics. There could be greater media promotion of mathematics as interesting and important. However, much more research is needed on the effectiveness of different strategies for improving attitudes to mathematics. In such research, it must be taken into account, both that mathematics has many components and that different strategies might be effective with different components; and that improving attitudes to mathematics means not only reducing anxiety and other negative emotions toward mathematics, but increasing positive emotions toward mathematics.

Treatments of already-established mathematics anxiety may involve both mathematics interventions as such, and treatments for anxiety such as systematic desensitization and cognitive behavior therapy. So far, no miracle cure seems to be in sight. However, there are new methods, based on recent research findings that appear to be promising.

In particular, researchers have recently attempted to use findings about the cognitive aspects of mathematics anxiety, and about cognitive treatments of anxiety more generally, to develop techniques involving reappraisal of the anxiety-provoking situation. A few recent studies suggest that instructing people to reappraise the nature and consequences of mathematics anxiety may reduce the negative effects, breaking a vicious circle, whereby people feel that their anxiety will worsen their performance or is a signal of inability to carry out the tasks. Johns et al. (2008) and Jamieson et al. (2010) found that informing people that arousal could actually improve performance led to better mathematics performance than in a control condition.

Beilock and colleagues have developed a promising intervention for mathematics anxiety that amounts to “writing out” the negative affect and worry (Ramirez and Beilock, 2011; Park et al., 2014). The researchers drew on previous findings that writing about traumatic and highly emotional events lowered ruminating behavior in individuals with clinical depression (Smyth, 1998). A possible mechanism for this could be that writing enables a form of reappraisal that interrogates the need to worry in the first place. This in turn frees working memory resources consumed by worrying, which can be deployed toward task performance. Ramirez and Beilock (2011) tested this proposition both in a laboratory environment and also in a high-stakes field experiment (i.e., an exam). Both the laboratory and field experiments showed that writing about one's worries before academic performance significantly improved performance compared to a control condition (e.g., writing about untested exam material). An exam can be stressful for anyone taking it. Most interesting, therefore, was the finding that 10 min of expressive writing before an exam was only beneficial for individuals with high test anxiety, compared to control writing. Individuals with low test anxiety did not experience any particular benefits from expressive writing. The authors attribute this to the extent to which individuals with high and low test anxiety differ in worrying about exams. Individuals with lower test anxiety, who presumably worry less, would therefore write about fewer worries during an expressive writing exercise. In other words, there is simply less worry that needs to be “written out” for individuals with low test anxiety, in contrast to individuals with high mathematics anxiety. The potential of this kind of intervention to facilitate a level playing field during exams is potentially large. Indeed, students in the expressive condition outperformed those in the control condition by 6%. In letter grades, the expressive condition students earned a B+ on average, while those in the control condition earned a B–. Could this kind of intervention be useful for mathematics anxiety? The same group of authors has suggested that this may be the case. In a recent paper, Park et al. (2014) explored the influence of expressive writing on the link between mathematics anxiety and mathematics performance. Parallel to the Ramirez and Beilock (2011) results, Park et al. (2014) found that expressive writing ameliorated performance on tasks of modular arithmetic (specially developed working memory-intensive mathematics problems) in high mathematics anxiety individuals compared to a control writing task. As stated earlier in this paper, one of the central tenets of current theories of mathematics anxiety is that the negative emotional state and associated ruminations absorb *working memory* resources necessary for task completion. Expressive writing seems to disrupt the negative emotional cognitions, and allows individuals to engage with the mathematical tasks rather than the attendant anxiety. Unlike Ramirez and Beilock (2011), Park et al. (2014) did not test these propositions in the field with an actual mathematics exam. Therefore, the benefit of expressive writing on mathematics examination performance remains a presumption in need of verification. However, a note of cautious optimism is permissible, given both the promising results from the earlier field experiments as well as evidence of higher performance on working memory-intensive problems reported in Park et al. (2014). Future research can easily investigate this possibility, as the only requirement is that proctors instruct students to engage in a writing task 10 min before the start of an exam.

Recently, the potential of cognitive tutoring to intervene with mathematics anxiety has been explored. Supekar et al. (2015) examined whether an intensive, 8-week one-on-one math-tutoring programme, MathWise that was developed by Fuchs et al. (2013) to improve mathematical skills could remediate math anxiety of children aged 7–9 years old. Children underwent three sessions of 40–50 min mathematics tutoring per week. They reported math anxiety levels using the Scale for Early Mathematics Anxiety (Wu et al., 2012) and were scanned using fMRI before and after training. During scanning, children performed on an arithmetic problem-solving task (Addition task) and number-identification (Control task). This study found that tutoring reduced math anxiety scores and remediated aberrant functional responses and connectivity in emotion-related circuits associated with the basolateral amygdala in children with high mathematics anxiety, but not those with low mathematics anxiety. In particular, they found that children with greater tutoring-associated decreases in their amygdala activity showed higher reductions in mathematics anxiety. The authors proposed that similar to models of exposure-based therapy for anxiety disorders, sustained exposure to mathematical stimuli could reduce mathematics anxiety, possibly through modulating the role of the amygdala. Together, this study showed that a relatively short and intensive one-on-one cognitive tutoring could remediate mathematics anxiety through modulation of neural functions.

As highlighted by Sokolowski and Necka (2016) however, interpretations of these findings should consider that since children were categorized through the extreme group approach (into high or low math-anxious using a median-split of pre-test SEMA scores) and were not recruited on the basis of their math anxiety levels, it is possible that children with nearly average SEMA scores might have been included in the high math anxious group (which is typically defined, for example by Ashcraft and Kirk (2001), as the highest 20% of this population). Such classification might affect the interpretations of “aberrant neural responses” attributed to children with high mathematics anxiety. Nonetheless, Supekar et al. (2015) provided a proof-of-concept that behavioral interventions with simultaneous neural, social and cognitive assessments could contribute to our understanding of the relationship between individual differences and efficacy of interventions.

Another potential form of treatment, which is just beginning to be explored, involves non-invasive brain stimulation. Non-invasive brain stimulation techniques are used by researchers to modulate neural activity on broad areas of the cortex. Transcranial electrical stimulation (tES) has emerged as a painless technique in which mild electrical currents are applied to the scalp and can be used to both upregulate and downregulate neuronal activity underneath the cortex.

Might such a technique be useful as an intervention for mathematics anxiety? As stated above, some brain imaging research has examined the neurophysiological signatures of mathematics anxiety. These include abnormal amygdala activation (Young et al., 2012) associated with fear processing, activation of the dorsoposterior insula, associated with pain perception (Lyons and Beilock, 2012a), and hypoactivation of regions in the frontoparietal network such as the dorsolateral prefrontal cortex, associated with both cognitive control of negative emotions and with mathematical performance (Lyons and Beilock, 2012b). Transcranial electrical stimulation enables researchers to modulate cortical activity in regions that may facilitate greater emotional control over the negative emotional response to mathematical stimuli, thereby improving performance. Transcranial direct current stimulation (tDCS) is the most widely used form of tES. tDCS is a non-invasive and painless neuromodulation technique wherein a low direct current, usually between 1 and 2 mA, is transmitted into cortical tissue through scalp-electrodes (Nitsche et al., 2008; Cohen Kadosh, 2013; Krause and Cohen Kadosh, 2014). The electrical signals in tDCS alter neuronal polarization, thereby manipulating the probability that the targeted neurons will fire; typically, anodal stimulation is known to facilitate neural firing, while cathodal stimulation inhibits neuronal firing of the stimulated cortical region (Nitsche and Paulus, 2000). In sham (placebo) stimulation, a burst of current is provided and turned-off, generating the same physical sensations as real stimulation (e.g., mild itching, burning, tingling, or stinging), but producing no change in cortical excitability. This serves as a reliable blinding method, and participants are generally unable to distinguish between real and sham stimulation (Gandiga et al., 2006). The brain region usually targeted in emotion-related tDCS research is the dorsolateral prefrontal cortex (dlPFC), which is implicated in working memory and affective regulation (Peña-Gómez et al., 2011), and is closely involved in the response and control of stress (Cerqueira et al., 2008).

Sarkar et al. (2014) investigated the effects of tDCS to the dlPFC on mathematics anxiety. High mathematics anxiety individuals received 1 mA of tDCS for 30 min (or 30 s, in the placebo condition) to their left and right dorsolateral prefrontal cortices to enhance cognitive control over the negative emotional response elicited by mathematical stimuli. A low mathematics anxiety group received the same treatment. Sarkar et al. (2014) also examined changes in salivary cortisol, mentioned above as a possible physiological measure of anxiety. Anodal and cathodal stimulation were applied to the left and right dlPFC, respectively. In their study, Sarkar et al. (2014) found that, compared to sham stimulation, real tDCS lowered reaction times in the arithmetic decision task for individuals with high mathematics anxiety. They found the opposite pattern for low mathematics anxiety participants, who were slower in real compared to sham stimulation. The cortisol changes mirrored the behavioral changes. Compared to sham stimulation, high mathematics anxiety participants showed a decline in salivary cortisol concentrations from pre- to post-test during real tDCS. For the low mathematics anxiety group, salivary cortisol concentrations declined from pre-test to post-test only during sham tDCS, but not during real stimulation. This suggests tDCS might be able to alleviate the stress associated with mathematics anxiety, thereby improving mathematical performance in individuals with high mathematics anxiety. It is still necessary to be cautious about this possibility for several reasons. Firstly, as discussed above, the relationship between cortisol secretion and mathematics anxiety may not be totally straightforward. Secondly, the ecological validity of such intervention (e.g., as regards the training design and the practicality of using tDCS outside the laboratory) remains to be improved (Cohen Kadosh, 2014; Looi et al., 2016). In the context of mathematics anxiety, further research is needed to examine whether tDCS could enhance performance for individuals with high mathematics anxiety in real-life settings and examinations (e.g., high-stakes situations). Given that the arithmetic decision task used by Sarkar et al. (2014) only required participants to decide whether very basic mathematical equations were true or false (e.g., 8 × 2 = 16, true or false), future studies could adopt more complex, realistic tasks. Thirdly, the improvement on such tasks was to the degree of ~50 ms, significant in a laboratory context but hardly relevant to the types of situations where mathematics anxiety is most relevant. Since behavioral studies mostly observe the influence of mathematics anxiety on difficult maths tasks (see Artemenko et al., 2015 for a recent review) and tES appears to be more effective during difficult tasks (Popescu et al., 2016), future studies could investigate whether improvements of individuals with mathematics anxiety would be greater during more difficult tasks. Fourthly, since the dlPFC is involved in many functions, it is as yet unclear exactly which of these functions was crucially affected here: in particular, whether tDCS affected performance by influencing its role in emotional processing, or working memory, or both. Fifthly, the findings suggest that such treatments would need to be targeted to people who are high in mathematics anxiety, and that their indiscriminate application to people with lower mathematics anxiety might actually impair performance. Hence, research that examines the mechanisms of such effects (positive or negative; short- or long-term) is needed (Bestmann et al., 2015). Finally, behavioral effects are influenced by the parameters of tDCS. For example, while Sarkar et al. (2014) showed that tDCS applied during mathematical tasks benefited those with high mathematics anxiety and impaired performance of those with low mathematics anxiety, it remains to be investigated whether changing the parameters of stimulation (e.g., applying stimulation before or after mathematical tasks) would yield different behavioral outcomes (for a review of other factors, see Looi and Cohen Kadosh, 2015). Thus, these findings are merely the first, though a promising step in the development of tES as a potential intervention for mathematics anxiety.

## So what remains to be understood?

During the last 60 years, we have acquired a much greater understanding of the phenomenon of mathematics anxiety. We have learned more about its correlation with mathematics performance, and for example how working memory may be involved in this. We have learned more about how it changes with age. We have learned more about its relationship to social stereotypes, especially with regard to gender. We have learned something about neural correlates of mathematics anxiety. We have learned something about possible ways to treat mathematics anxiety.

Thus, we have learned a significant amount about many specific aspects of mathematics anxiety. Our biggest need for further learning may involve not so much any specific aspect, as the ways in which the aspects relate to one another. How do the social aspects relate to the neural aspects? How do either or both of these relate to changes with age? How might appropriate treatment be related to age and to the social and cognitive characteristics of the individuals? And of course the perennial “chicken and egg” question: does mathematics anxiety lead to poorer performance, or does poor performance, with its resulting experiences of failure, lead to poorer performance (Carey et al., 2015)? Many more interdisciplinary, longitudinal and intervention studies will be needed to answer these questions. An ultimate goal of such research is to integrate findings from across the behavioral, cognitive and biological dimensions of this construct in order to produce a fuller description of mathematics anxiety as a trait that varies between individuals.

There are also more specific aspects of mathematics anxiety that need a lot more study. For example, although there has been a great deal of research on social influences on mathematics anxiety, most of this has involved one particular type of influence: gender stereotyping. Other influences also need more investigation. In particular, there needs to be more investigation of the role of pressures by parents and teachers for school achievement. This is especially true in view of the increasing importance of both mathematics as such and of academic qualifications in today's society; and in view of the increasing concern of governments in several countries about raising academic standards. The question arises of whether and at what point an increasing emphasis on mathematical achievement might have the negative and potentially counterproductive effect of increasing mathematics anxiety; and how this might be prevented. In this context, there needs to be more research on exactly how mathematics anxiety is related to motivation, and, in particular, whether there are differences in the relationships of intrinsic and extrinsic motivation to anxiety (Gottfried, 1982; Lepper, 1988; Ryan and Pintrich, 1997).

We hope that long before another 60 years have passed, research will have led to a greater understanding of mathematics anxiety, which will enable us to develop interventions and educational methods that will greatly reduce its incidence.

## Author contributions

All authors listed, have made substantial, direct, and intellectual contribution to the work, and approved it for publication.

## Funding

We thank the Nuffield Foundation for financial support.

### Conflict of interest statement

The authors declare that the research was conducted in the absence of any commercial or financial relationships that could be construed as a potential conflict of interest.
